# Outbreak of Antiviral Drug–Resistant Influenza A in Long-Term Care Facility, Illinois, USA, 2008

**DOI:** 10.3201/eid1512.081644

**Published:** 2009-12

**Authors:** Nila J. Dharan, Monica Patton, Alicia M. Siston, Julie Morita, Enrique Ramirez, Teresa R. Wallis, Varough Deyde, Larisa V. Gubareva, Alexander I. Klimov, Joseph S. Bresee, Alicia M. Fry

**Affiliations:** Centers for Disease Control and Prevention, Atlanta, Georgia, USA (N.J. Dharan, M. Patton, T.W. Wallis, V. Deyde, L.V. Gubareva, A.I. Klimov, J.S. Bresee, A.M. Fry); Chicago Department of Public Health, Chicago, Illinois, USA (A.M. Siston, J. Morita, E. Ramirez); 1 Current affiliation: New York University School of Medicine, New York, New York, USA.; 2 Current affiliation: Centers for Disease Control and Prevention, Atlanta, Georgia, USA.

**Keywords:** Influenza, outbreak, oseltamivir, drug resistance, viruses, nursing homes, infection control, Illinois, expedited, dispatch

## Abstract

An outbreak of oseltamivir-resistant influenza A (H1N1) occurred in a long-term care facility. Eight (47%) of 17 and 1 (6%) of 16 residents in 2 wards had oseltamivir-resistant influenza A virus (H1N1) infections. Initial outbreak response included treatment and prophylaxis with oseltamivir. The outbreak abated, likely because of infection control measures.

Outbreaks of influenza virus infection cause illness and death, especially among residents of long-term care facilities (LTCFs). In addition to annual vaccination and infection control measures, antiviral agents for treatment and prophylaxis are useful components for control of influenza outbreaks in LTCFs (*1*–*4*), especially in years with vaccine strain mismatches (*4*).

Two classes of antiviral agents are licensed for use in the United States: adamantanes (amantadine and rimantadine) and neuraminidase inhibitors (oseltamivir and zanamivir). Circulation of influenza A viruses resistant to both classes of antiviral agents, A (H3N2) to adamantanes and A (H1N1) to oseltamivir, was reported during the 2007–08 influenza season (*5*). We describe an outbreak of illness in an LTCF caused by 2 influenza viruses, an oseltamivir-resistant A virus (H1N1) and an adamantane-resistant A virus (H3N2), during January 2008.

## The Study

The LTCF in Illinois provides housing, healthcare services, and recreational activities for residents with neurologic and developmental medical conditions. During the outbreak, the LTCF housed 583 residents. Building A, the main site of the influenza outbreak, housed 108 residents in 6 wards; 104 (96%) received the 2007–08 influenza vaccine. Of the 685 LTCF employees involved in direct patient care, 385 (56%) received the 2007–08 influenza vaccine on site.

We defined a confirmed case as a positive rapid or reverse transcription–PCR result for influenza virus from January 20 through February 8, 2008, in a resident of the LTCF. Surveillance for new case-patients included obtaining a nasopharyngeal specimen from all residents with new onset of fever or respiratory symptoms or any unusual behavior within 24 hours after illness onset. All specimens were tested by using the QuickVue A and B Influenza Test (Quidel, San Diego, CA, USA). A second specimen was obtained from all persons with positive rapid test results and some (57%) from persons with negative results for confirmation of influenza virus infection and virus subtyping by reverse transcription–PCR. Medical records, vaccination records, resident activity, and visitor logs were reviewed.

Testing for antiviral drug resistance was conducted directly on clinical specimens by pyrosequencing as described (*6*,*7*), including identification of the oseltamivir resistance–conferring H274Y mutation in the neuraminidase gene of influenza viruses (H1N1) (H275Y in N1 numbering) and the adamantane resistance–conferring mutations in the matrix 2 protein (*7*,*8*). The HA1 portion of the hemagglutinin (HA) gene of the outbreak viruses was sequenced and compared with those of epidemiologically relevant viruses.

Phylogenetic analysis of HA1 was performed by using MEGA version 4.0.1 software (*9*). A phylogenetic tree was inferred by using maximum composite likelihood available in MEGA version 4.0.1. The outbreak investigation was considered a public health response and granted exemption from review by the Institutional Review Board of the Centers for Disease Control and Prevention.

**Figure 1:**
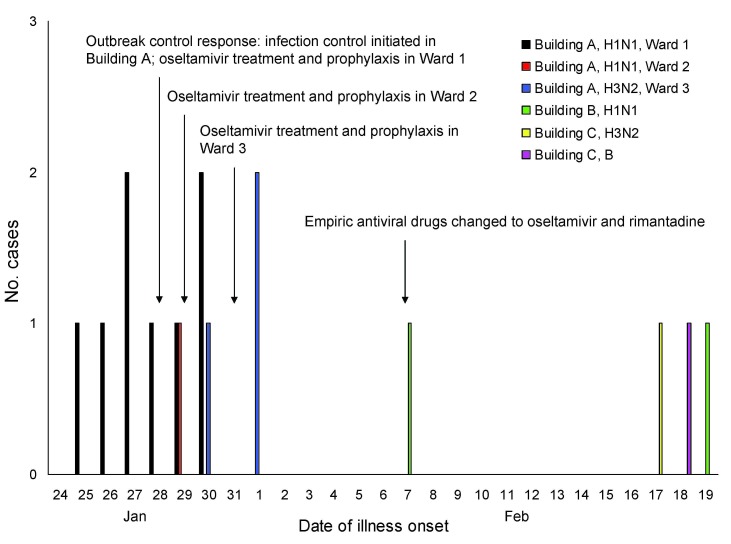
Number of cases of influenza by date of symptom onset and outbreak control protocol during an influenza A outbreak in a long-term care facility, Illinois, USA, 2008. Retrospective medical chart review of all nontested building A residents identified 1 potential missed case-patient with influenza who had symptom onset on January 29. Additional cases were detected in 2 other residential buildings in the long-term care facility (buildings B and C). Building B housed 53 residents in 4 wards and building C housed 16 residents in 1 ward. All (100%) of residents in both buildings had received the 2007–08 influenza vaccine. Of the 16 rapid test specimens with negative results from all 3 buildings that were subjected to confirmatory testing, 5 (31%) were positive by reverse transcription–PCR for influenza A virus (H1N1).

On January 27, the first 3 residents with fever or respiratory symptoms in ward 1 within building A were positive for influenza A virus infection by rapid test (Figure 1). On January 28, outbreak infection control measures were initiated in all 6 wards, including surveillance for new cases, 5 days of treatment with oseltamivir for confirmed cases, and 14 days of prophylaxis with oseltamivir for all healthy residents in wards with confirmed case-patients (*2*). Confirmed case-patients were quarantined in their rooms for 10 days; all residents in all 6 wards were quarantined for 10 days, and visitor movement was restricted. Staff and visitors were required to use personal protective equipment and practice respiratory and hand hygiene. Prescriptions for prophylactic courses of oseltamivir and influenza vaccinations were offered to all staff of building A; uptake was not recorded.

From January 28 through January 31, 2008, a total of 6 additional confirmed case-patients were identified. Eight (47%) of 17 residents in ward 1 and 1 (6%) of 16 residents in ward 2 were infected with influenza A viruses (H1N1) that contained the H274Y mutation but did not have markers of resistance to adamantanes or zanamivir.

On January 30, high fever developed in a male resident in ward 3 while on the first day of a home visit (Figure 1). He returned to building A on January 31, was positive for influenza by rapid test, and was placed in ward 2 in an attempt to group him with other already ill residents. Because of an ongoing outbreak in other nearby wards, oseltamivir prophylaxis was initiated for all residents in ward 3 who were not ill. On February 1, symptoms developed in 2 other residents in ward 3 who were positive for influenza by rapid test. Three (18%) confirmed cases of influenza A virus (H3N2) resistant to adamantanes but sensitive to oseltamivir were detected among 17 residents in ward 3. Additional cases, but no clusters, were detected in other buildings 1–2 weeks later.

Characteristics of case-patients are shown in the Table. Establishing a firm epidemiologic link between cases, other than ward of residency, was not possible. Antiviral drug resistance results became available on February 7 when all case-patients had completed their treatment courses. Ongoing prophylaxis courses were changed: oseltamivir was replaced with rimantadine in ward 1, and rimantadine was added to oseltamivir in ward 2. Prophylaxis with oseltamivir alone was continued in ward 3. Zanamivir could not be used by most residents because of underlying conditions.

**Table:** Characteristics of 12 confirmed influenza case-patients in building A, long-term care facility, Illinois, USA, 2008*

Characteristic	Influenza virus subtype	
	A (H1) (n = 9)	A (H3) (n = 3)
Age, y, median (range)	29 (14–47)	32 (21–37)
Any underlying medical conditions	9 (100)	3 (100)
Neurologic disorders	9 (100)	3 (100)
Gastrointestinal disorders	8 (89)	3 (100)
Pulmonary disease	2 (22)	2 (67)
Fever >100.5°F	9 (100)	3 (100)
Cough	7 (78)	2 (67)
Desaturation	4 (44)	1 (33)
Lowest % oxygen saturation, median (range)	88.5 (88–92)	92 (NA)
Elevated or new oxygen requirement	2 (22)	0
Difficulty breathing	2 (22)	1 (33)
Increased secretions	3 (33)	2 (67)
Increased respiratory rate	3 (33)	0
Lethargy	1 (11)	0
Distress	3 (33)	0
Elevated level of care	4 (44)	1 (33)
Hospitalized†	1 (11)	0
Length of stay, d	1	NA
Clinical treatment		
Antimicrobial drugs	3 (33)	0
Antipyretics	9 (100)	3 (100)
Nebulizer (albuterol)	3 (33)	1 (33)
Received 2007–08 influenza vaccine	8 (89)	3 (100)
Died‡	1 (11)	0

*All values are no. (%) case-patients except as indicated. NA, not applicable. †One case-patient was hospitalized for parotitis to rule out infection with mumps and was discharged in stable condition after 1 day. ‡One case-patient who had a diagnosis of end-stage lung disease and a do not resuscitate/do not intubate directive died.

**Figure 2:**
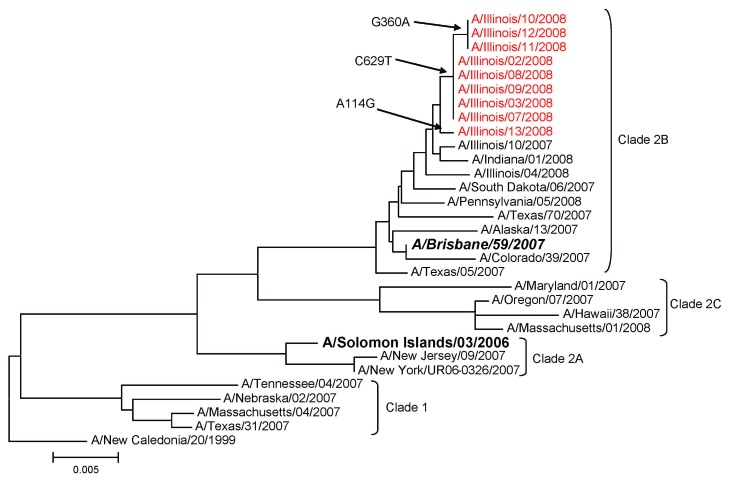
Phylogenetic analysis of the hemagglutinin gene (HA1 portion) of influenza A viruses (H1N1) isolated during an influenza A outbreak in a long-term care facility, Illinois, USA, 2008. Viruses from buildings A and B shared nearly identical sequences. One of the viruses from building B was more similar in sequence to 1 virus from building A. However, this finding could reflect natural variance in circulating viruses. Red indicates outbreak viruses, ***boldface italics*** indicates vaccine strain for 2008–09, **boldface** indicates vaccine strain for 2007–08, and arrows indicate nucleotide differences in HA1 subunit. Scale bar indicates nucleotide substitutions per site.

Sequence analysis of the HA1 gene in outbreak influenza A viruses (H1N1) showed identical or nearly identical sequences, differing by only 1 or 2 nt (Figure 2). These viruses were phylogenetically more closely related to A/Brisbane/59/2007 (H1N1) than to the A/Solomon Islands/3/2006, the influenza A virus (H1N1) strain in the 2007–08 influenza vaccine. GenBank accession numbers of HA (HA1) sequences for the 9 oseltamivir-resistant influenza A viruses (H1N1) are FJ231752–FJ231760.

## Conclusions

The attack rate of illness caused by oseltamivir-resistant influenza A viruses (H1N1) in ward 1 was within the range (20%–80%) reported for other facility influenza outbreaks (*1*,*10*,*11*), indicating effective person-to-person transmission of oseltamivir-resistant influenza A viruses (H1N1). Nosocomial transmission of oseltamivir-resistant influenza A viruses (H1N1), with possible healthcare worker involvement, has been described (*12*). We were unable to assess staff illness in this investigation. Before the 2007–08 influenza season, transmission of neuraminidase-resistant influenza viruses had rarely been reported (*13*).

Although we documented a relatively high attack rate in 1 ward (ward 1), and despite resistance to the antiviral agent initially used, the outbreak abated quickly. High annual vaccination rates among residents and relatively high rates among employees (*2*) may have played a role in limiting the spread of the outbreak viruses. However, the A/Brisbane/59/2007 (H1N1)–like outbreak viruses were not optimally matched to the A/Solomon Islands/3/2006 (H1N1) vaccine strain (*14*). Also, infection control measures, such as isolation and quarantine, likely played a role in controlling this outbreak.

The proportion of circulating influenza viruses resistant to oseltamivir increased from 12% during the 2007–08 season to 99% during the 2008–09 season in the United States, and new interim guidelines for use of antiviral agents were released in December 2008 (*15*). These guidelines were updated for the 2009–10 season to account for the emergence of pandemic (H1N1) 2009 virus in September 2009 (www.cdc.gov/h1n1flu/recommendations.htm). This outbreak underscores the possibility of 2 influenza A viruses, with different antiviral susceptibilities, in the same facility. During a facility outbreak of influenza, providers should consult antiviral recommendations of the Centers for Disease Control and Prevention and obtain influenza virus typing and subtyping to guide appropriate antiviral drug choices.
